# Baseline CD4 and mortality trends in the South African human immunodeficiency virus programme: Analysis of routine data

**DOI:** 10.4102/sajhivmed.v20i1.963

**Published:** 2019-07-24

**Authors:** Rivka R. Lilian, Kate Rees, Moyahabo Mabitsi, James A. McIntyre, Helen E. Struthers, Remco P.H. Peters

**Affiliations:** 1 Anova Health Institute, Johannesburg, South Africa; 2 School of Public Health and Family Medicine, University of Cape Town, Cape Town, South Africa; 3 Division of Infectious Diseases and HIV Medicine, Department of Medicine, University of Cape Town, Cape Town, South Africa; 4 School of Public Health, University of the Witwatersrand, Johannesburg, South Africa; 5 Department of Medical Microbiology, School of Public Health and Primary Care (CAPHRI), Maastricht University Medical Centre, Maastricht, the Netherlands

**Keywords:** TIER.Net, Antiretroviral Therapy, CD4, Mortality, HIV, South Africa

## Abstract

**Background:**

Despite widespread availability of antiretroviral therapy (ART) in South Africa, there remains a considerable burden of human immunodeficiency virus (HIV)-related morbidity and mortality.

**Objectives:**

To describe ART initiation and outcome trends over time, with a focus on clients presenting with advanced HIV-infection, so as to identify interventions to reduce morbidity and mortality.

**Methods:**

Routine TIER.Net data from HIV-infected adults who had a documented baseline CD4 count and were newly initiating ART in Johannesburg or Mopani districts from 2004 to 2017 were analysed. Trends in baseline CD4 count and 5-year mortality were investigated and the population initiating ART with CD4 < 200 cells/mm^3^ was described.

**Results:**

The Johannesburg and Mopani data sets comprised 203 131 and 101 814 records, respectively. Although median CD4 count increased over time, the proportion of initiations at CD4 < 200 cells/mm^3^ in 2017 remained high (Johannesburg 39%, Mopani 35%). Mortality was significantly increased among clients with CD4 < 200 compared to those with higher baseline counts (*p* < 0.001). Even though mortality among clients with low CD4 declined over time, likely because of improved drug regimens, in 2016–2017 mortality was still significantly increased among these clients (*p* < 0.001). Delivery of cotrimoxazole prophylaxis to clients with low CD4 declined over time to < 30% in 2017 and was associated with clinical stage. Presentation with CD4 < 200 cells/mm^3^ was associated with older age, male gender and hospitalisation.

**Conclusion:**

A concerningly large proportion of South Africans still initiate ART at low CD4 counts. This is associated with increased mortality and requires targeted interventions to improve delivery of prophylactic regimens and early engagement in care.

## Introduction

In 2017, there were 6.9 million adults living with human immunodeficiency virus (HIV) in South Africa, of whom 61% were receiving treatment.^1^ This equates to 4.2 million adults receiving antiretroviral therapy (ART), forming the largest treatment programme in the world.^1^ South Africa’s ART programme has demonstrated successful initiation and management of clients on treatment for over a decade, despite rapid programmatic scale-up over this time,^2^ and has achieved near-normal life expectancy for HIV-infected adults with timely ART initiation.^3^ The ART programme is thus an essential component of the third Sustainable Development Goal which aims to ensure healthy lives and end the AIDS epidemic by 2030.^4^

South Africa’s ART programme has evolved over time to accommodate increasing numbers of HIV-infected clients and to provide improved care and treatment services (Table 1).^5,6,7,8,9,10^ This has encompassed expansion of the ART eligibility criteria from clients with CD4 counts < 200 cells/mm^3^ in 2004^5^ to universal test and treat (UTT) in 2016,^8^ as well as a shift from doctor- to nurse-managed care and decentralisation of ART services from hospitals to primary healthcare sites.^11^ Across all years, cotrimoxazole preventive therapy (CPT) has been recommended for clients with baseline CD4 counts below 200 cells/mm^3^ as a means of protecting against infections and thereby reducing morbidity and mortality.^5,6,9^ Triple-therapy antiretroviral drug regimens have also been updated over time, from combination single formulation regimens including stavudine in 2004^5^ and tenofovir in 2010^6^ to a fixed-dose combination (FDC), a single tablet containing three antiretroviral drugs (tenofovir, emtricitabine and efavirenz), in 2013.^7^ As the ART programme has evolved and expanded, it has become increasingly important to have an effective monitoring system, and in 2010 the Department of Health adopted an electronic monitoring and evaluation tool known as TIER.Net, which was developed by the University of Cape Town’s Centre for Infectious Disease Epidemiology and Research.^12^ TIER.Net is used operationally to monitor baseline clinical care and client outcomes over time, providing a rich source of cross-sectional and longitudinal routine ART data.

**TABLE 1:** South African guidelines for treatment of adults with human immunodeficiency virus infection.

Variable	2004 guidelines	April 2010 guidelines	March 2013 guidelines†	December 2014 guidelines‡	August 2016 circular‡
ART eligibility	CD4 count < 200 cells/mm^3^ *or* WHO Stage IV disease	CD4 count ≤ 200 cells/mm^3^ *or* CD4 count ≤ 350 cells/mm^3^ in clients with TB/HIV or pregnant women *or* WHO stage IV disease *or* MDR/XDR-TB	CD4 count ≤350 cells/mm^3^¶ *or* WHO stage III or IV disease *or* Clients with all types of TB	CD4 count ≤ 500 cells/mm^3^ *or* WHO stage III or IV disease *or* Active TB disease *or* Pregnant and breastfeeding women *or* Known HBV co-infection	UTT: all HIV-infected clients regardless of CD4 count
First-line ART regimen (new clients)	d4T + 3TC + EFV/NVP	TDF + 3TC/FTC + EFV/NVP	FDC††	FDC††	
CPT	All clients initiating ART	CD4 ≤ 200 cells/mm^3^ WHO stage II, III or IV disease (including TB)		CD4 count ≤ 200 cells/mm^3^ WHO stage III or IV disease HIV/TB co-infection	

*Source:* National Department of Health of South Africa^5,6,7,8,9^

3TC, lamivudine; ART, antiretroviral therapy; CPT, cotrimoxazole preventive therapy; d4T, stavudine; EFV, efavirenz; FDC, fixed-dose combination; FTC, emtricitabine; HIV, human immunodeficiency virus; HBV, hepatitis B; MDR/XDR-TB, multidrug-resistant or extensively drug-resistant tuberculosis; NVP, nevirapine; TB, tuberculosis; TDF, tenofovir disoproxil fumarate; UTT, universal test and treat; WHO, World Health Organization

† , Implementation date = 01 April 2013;

‡ ,Implementation date = 01 January 2015;

‡ , Implementation date = 01 September 2016;

¶ , Implementation of the CD4 count ≤350 cell/mm^3^ cut-off occurred in August 2011, prior to the publication of the 2013 guidelines^10^;

†† , FDC consists of TDF, FTC and EFV.

Despite the widespread availability of ART, there is still a considerable burden of HIV-related morbidity and mortality. In South Africa, HIV accounted for almost two-thirds of medical admissions at one hospital in 2012 and 2013,^13^ with no improvement in the number of deaths due to AIDS nationally from 2013 to 2017,^14^ and in West Africa, AIDS-defining conditions remained the primary cause of hospitalisation among HIV-infected adults years after the scale-up of ART services.^15^ High morbidity and mortality is specifically associated with late presentation for HIV care, as indicated by low baseline CD4 counts or advanced clinical stage.^3,16,17,18^ These poor outcomes potentially undermine the population-level impact of the ART programme, as ART coverage in individuals is known to impact HIV transmission, incidence and community viral load.^19,20^ Interventions to improve ART initiation, specifically among clients at risk of presenting late for HIV care, are therefore essential to improve both individual- and programme-level outcomes. This study used operational programme data to describe ART initiation and outcome trends over time, with a focus on clients presenting late for care, so as to identify programmatic gaps that can guide interventions to reduce HIV-associated morbidity and mortality. Two districts were investigated as examples of the urban and rural HIV epidemics in South Africa, namely, Johannesburg and Mopani districts. Specifically, this study aims to use routine TIER.Net data from adult clients in an urban and rural district of South Africa to (1) describe ART programme growth and baseline CD4 count over time, (2) analyse 5-year mortality in the context of baseline CD4 count and (3) describe the population initiating ART at low CD4 counts (< 200 cells/mm^3^) in 2017 in order to identify priority groups at high risk of mortality for intervention.

## Methods

### Study population and data source

Routinely collected data from adults initiating ART in two districts of South Africa, Johannesburg district in Gauteng province and Mopani district in Limpopo province, were analysed. Of the seven regions in Johannesburg, four (C, D, E and G) were included in the analysis, as these regions have been supported by Anova Health Institute and routine data were therefore available for analysis. Johannesburg district has a population density of 3044 persons/km^2^ and is relatively economically affluent, falling into socio-economic quintile 5.^21^ In contrast, Mopani district is sparsely populated and socio-economically deprived, having a population density of 56.9 persons/km^2^ and a socio-economic quintile of 2.^21^ Antenatal HIV prevalence, a proxy for overall population prevalence, is 29.6% and 24.5% in Johannesburg and Mopani districts, respectively.^22^ Both districts provide HIV care and treatment services, with 71.8% and 72.9% of adults diagnosed with HIV-infection being retained on ART at 12 months, respectively.^21^

In April 2018, data for Johannesburg and Mopani districts were extracted from TIER.Net. Records were included in the analysis from clients initiating ART between 2004 and 2017 (inclusive), where clients were 15–80 years of age, were newly initiating ART and had a baseline CD4 count on record. In order to exclude outlying CD4 counts that were likely data errors, records with baseline counts above 2000 cells/mm^3^ were excluded. For the Kaplan–Meier analysis only, clients who were lost to follow-up (LTFU) or had transferred out of the ART programme were also excluded.

### Statistical analysis

Descriptive statistics were used to explore trends in ART initiation and baseline CD4 counts over time. Kaplan–Meier survival analysis was used to estimate the probability of death over time in clients with low baseline CD4 counts (< 200 cells/mm^3^). Only clients known to have died and those active in care were included in the Kaplan–Meier analysis. Follow-up time, defined as the time between ART start and last ART visit, was censored at 5 years after ART initiation. Where clients died after the initiation visit (i.e. ART initiation date and last visit date were the same), a follow-up time of half a day (0.001 years) was assigned. Survival curves were compared using a Peto–Peto–Prentice test for equality of survivor functions, which is not affected by differences in censoring patterns across groups and is appropriate even when hazard functions are not proportional.^23^

To describe the population presenting late for HIV care in 2017, clients with baseline CD4 counts < 200 cells/mm^3^ were compared to those with CD4 counts ≥ 200 cells/mm^3^ using Mann–Whitney and chi-squared (χ^2^) or Fisher’s exact tests for continuous and categorical variables, respectively. Viral load suppression was calculated using the last viral load test on record for each client and was defined as a last viral load result < 1000 copies/mL as per South African guidelines, which use 1000 copies/mL as the cut-off for virological failure.^9^ Delivery of CPT to clients presenting for HIV care with baseline CD4 counts < 200 cells/mm^3^ was compared between clients with advanced clinical disease (World Health Organization [WHO] stage III or IV disease) and clients with WHO stage I or II disease using 95% confidence intervals over the last 5 years (2013–2017). Analyses were performed using Microsoft Excel 2010 and Stata version 14.2 (StataCorp LLC, College Station, TX, USA). A *p-*value of < 0.05 was considered significant.

## Ethical consideration

The study was approved by the University of the Witwatersrand’s Medical Ethics Committee (M140461). Individual patient consent was not required as this study analysed anonymised TIER.Net data that were routinely collected at healthcare facilities for monitoring purposes. No data collection was performed for the purposes of this study. In addition, no patient files or electronic medical records were retrieved or accessed at any stage.

## Results

### Description of study population

The Johannesburg data set comprised 340 023 records from adult clients aged 15–80 years initiating ART between 2004 and 2017. Records from 83 677 (25%) clients were excluded, as these individuals were not newly initiating ART. A further 53 178 (16%) records were excluded because of missing baseline CD4 counts. Among the remaining 203 168 records, 37 (< 1%) had baseline CD4 counts above 2000 and thus were also excluded, leaving a Johannesburg data set of 203 131 records for analysis. The initial Mopani data set comprised 159 904 records from adults initiating ART between 2004 and 2017, of which 36 553 (23%) were excluded because of ART initiation in clients who were not treatment naïve and a further 21 517 (13%) were excluded because of missing baseline CD4 counts. Among the remaining 101 834 records, 20 (< 1%) with baseline CD4 counts above 2000 were also excluded, leaving a Mopani data set of 101 814 records for analysis.

### Antiretroviral therapy initiation and baseline CD4 counts over time

**FIGURE 1:**
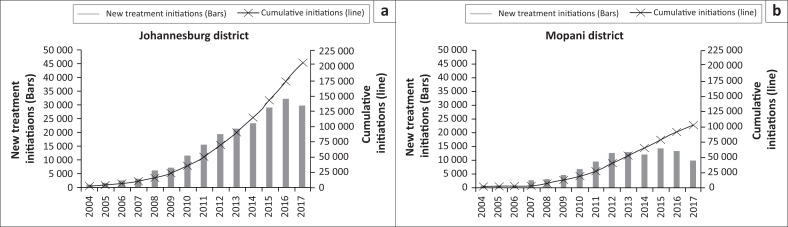
Antiretroviral therapy initiations over time among adult clients newly initiating treatment in (a) Johannesburg district and (b) Mopani district.

The ART programmes in both Johannesburg and Mopani districts expanded rapidly over time (Figure 1). A total of 203 131 adults were initiated on treatment in Johannesburg compared to 101 814 in Mopani. The number of ART initiations increased steadily each year from 2004 to 2013, but began to decline in Johannesburg from 2016 and in Mopani from 2015. Across all years, the majority of clients in both districts were women, ranging from 63% to 66% in Johannesburg and 68% to 77% in Mopani. Median age at ART initiation ranged from 35 to 37 years in Johannesburg and from 36 to 39 years in Mopani.

**FIGURE 2:**
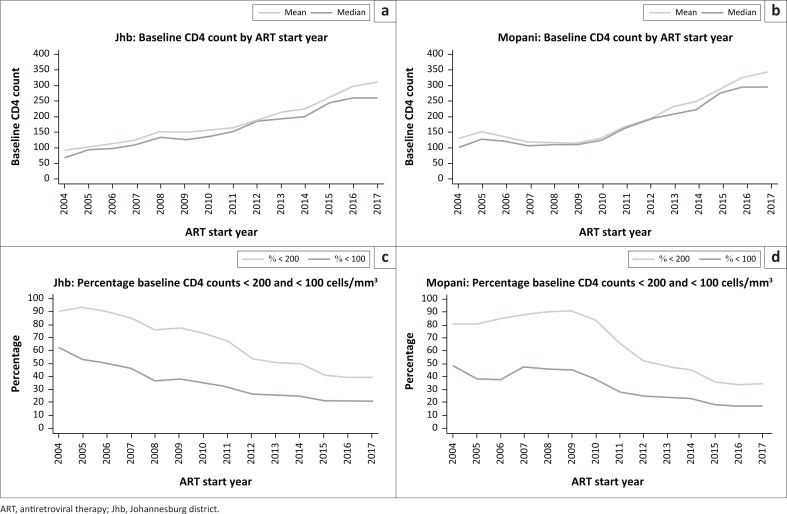
Mean and median baseline CD4 count over time and percentage baseline CD4 counts < 200 and < 100 cells/mm^3^ in Johannesburg district (a and c) and Mopani district (b and d).

The mean and median baseline CD4 counts increased from 2004 to 2017 in both Johannesburg and Mopani districts (Figure 2a and b). The increase has been consistent in Johannesburg, but Mopani took a longer time to show this trend, with a consistent increase only being evident from 2009. Although the proportion of baseline CD4 counts < 200 cells/mm^3^ has decreased over time, particularly from 2009, there has been no decrease in the last 2 years, with percentage counts < 200 cells/mm^3^ remaining at ± 40% in Johannesburg and ± 35% in Mopani (Figure 2c and d).

### Mortality survival analysis

**FIGURE 3:**
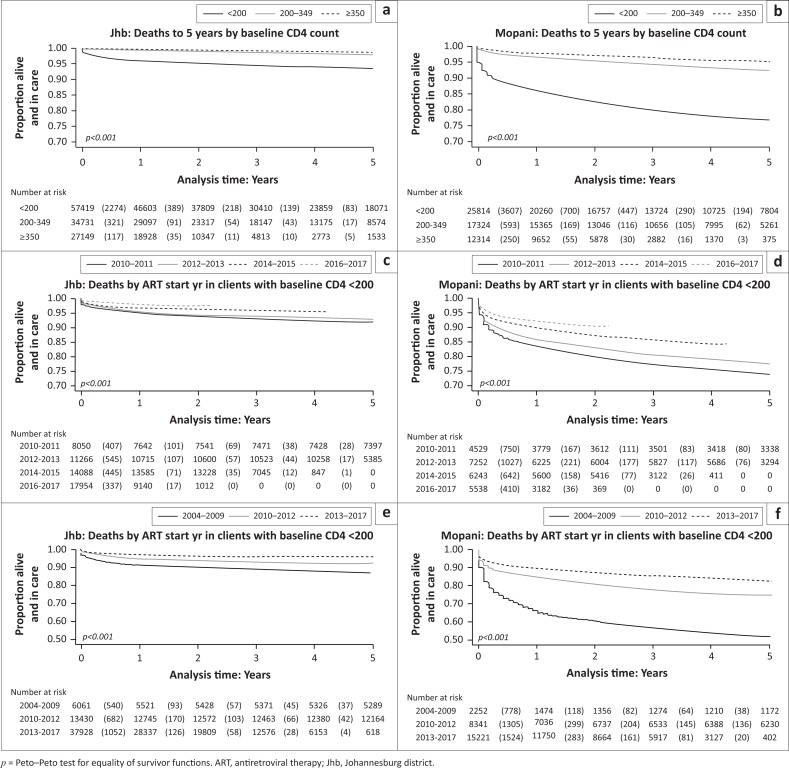
Kaplan–Meier survival curves of deaths to 5 years by baseline CD4 count in (a) Johannesburg and (b) Mopani. Deaths by year of antiretroviral therapy initiation among clients with baseline CD4 counts < 200 cells/mm^3^ in (c) Johannesburg and (d) Mopani. Deaths by year of antiretroviral therapy initiation among clients with baseline CD4 counts < 200 cells/mm^3^ where years have been grouped according to the timing of drug changes in the South African guidelines in (e) Johannesburg and (f) Mopani (2004–2009: single formulation regimens including stavudine; 2010–2012: single formulation regimens including tenofovir; 2013–2017: fixed-dose combination).

Mortality was significantly higher among clients who initiated ART with CD4 counts < 200 cells/mm^3^ compared to those with higher baseline CD4 counts, with 5-year death rates of 6%, 2% and 1% among clients with CD4 counts < 200, 200–349 and ≥ 350 cells/mm^3^, respectively, in Johannesburg and 23%, 7% and 5%, respectively, for the same CD4 counts in Mopani (*p* < 0.001 for both districts) (Figure 3a and b). Early deaths immediately after ART initiation among clients with baseline CD4 < 200 cells/mm^3^ are clearly evident. Even in recent years (2016 and 2017), clients with baseline CD4 < 200 cells/mm^3^ had significantly higher mortality than those with higher CD4 counts (losses of 2.4% and 0.4% in Johannesburg and 9% and 2% in Mopani among clients with CD4 counts < 200 and ≥ 200 cells/mm^3^, respectively (*p* < 0.001 for both districts)). Among clients with baseline CD4 < 200 cells/mm^3^, mortality was lower among those who initiated ART in recent years compared to those who initiated treatment in earlier years of the ART programme (*p* < 0.001 for both Johannesburg and Mopani) (Figure 3c and d). When ART initiation years were grouped according to timing of drug changes in the South African guidelines, clients with baseline CD4 < 200 cells/mm^3^ who initiated treatment in years when stavudine was in use had the highest mortality in both districts, with 5-year losses of 13% in Johannesburg and 48% in Mopani (Figure 3e and f). In both districts, clients who initiated treatment after tenofovir was introduced had lower mortality rates (5-year losses of 8% and 25%, respectively) and deaths were even lower after the introduction of an FDC (5-year losses of 4% and 17%, respectively).

### Characteristics and outcomes of clients initiating antiretroviral therapy at CD4 < 200 cells/mm^3^

Compared to clients with baseline CD4 counts ≥ 200 cells/mm^3^, clients who initiated ART in 2017 with low baseline CD4 counts were more likely to be men and to initiate treatment at older ages (*p* < 0.001) (Table 2). Clients with low baseline CD4 in both districts were more likely to initiate ART at a hospital compared to those starting ART with higher CD4 counts (*p* < 0.001). This correlates with findings of more advanced clinical stage in clients with low baseline CD4 (*p* < 0.001). Among women aged 15–50 years, a significantly lower percentage of clients with CD4 < 200 were pregnant during ART initiation compared to women with higher baseline counts in both districts (*p* < 0.001).

**TABLE 2:** Characteristics, care and outcomes of adult clients initiating antiretroviral therapy in 2017 by baseline CD4 count.

Variable	Johannesburg district					Mopani district				
	Baseline CD4 < 200		Baseline CD4 ≥ 200		*p*	Baseline CD4 < 200		Baseline CD4 ≥ 200		*p*
**CHARACTERISTICS OF CLIENTS AT BASELINE**										
**Gender**					**< 0.001**					**< 0.001**
Male	5255	45.2%	5294	29.3%		1347	40.4%	1471	23.3%	
Female	6375	54.8%	12 747	70.7%		1986	59.6%	4831	76.7%	
**Age at ART start, years (median, range)**	37.0	15.0–79.1	33.7	15.0–78.1	**< 0.001**	39.0	15.1–78.2	34.7	15.0–79.3	**< 0.001**
**Facility type**					**< 0.001**					**< 0.001**
Clinic	11199	96.3%	17 606	97.6%		3116	93.5%	6032	95.7%	
Hospital	431	3.7%	435	2.4%		217	6.5%	270	4.3%	
**WHO stage at ART start**					**< 0.001**					**< 0.001**
I	6044	54.7%	14 252	82.8%		2190	69.1%	5269	87.2%	
II	1838	16.7%	1877	10.9%		577	18.2%	610	10.1%	
III	2244	20.3%	901	5.2%		325	10.3%	142	2.4%	
IV	915	8.3%	175	1.0%		79	2.5%	22	0.4%	
**Pregnant at ART start, females aged 15–50 years**					**< 0.001**					**< 0.001**
Yes	927	16.6%	2918	25.4%		255	15.8%	1115	26.8%	
No	4666	83.4%	8560	74.6%		1358	84.2%	3049	73.2%	
**CARE OF CLIENTS AT BASELINE**										
**CPT at ART start**					**< 0.001**					**< 0.001**
Yes	2453	23.1%	1015	6.1%		784	25.5%	846	14.5%	
No	8156	76.9%	15 538	93.9%		2295	74.5%	5004	85.5%	
**IPT at ART start**					**< 0.001**					**0.037**
Yes	901	10.2%	1422	8.8%		709	25.6%	1605	27.7%	
No	7915	89.8%	14 747	91.2%		2063	74.4%	4186	72.3%	
**OUTCOMES OF CLINIC CLIENTS**										
**Mortality**					**< 0.001**					**< 0.001**
Active	9061	98.5%	14 533	99.8%		2333	95.0%	4591	98.9%	
Died	140	1.5%	32	0.2%		124	5.1%	51	1.1%	
**Programme outcome**					**< 0.001**					**0.019**
Active	9061	85.2%	14 533	86.8%		2333	80.3%	4591	82.4%	
Died or lost to follow-up	1572	14.8%	2214	13.2%		573	19.7%	983	17.6%	
**Suppression at last viral load test**					**< 0.001**					**< 0.001**
Suppressed	4092	79.0%	8107	89.4%		1202	80.7%	2818	87.5%	
Not suppressed	1090	21.0%	957	10.6%		287	19.3%	404	12.5%	
**OUTCOMES OF HOSPITAL CLIENTS**										
**Mortality**					0.099					**< 0.001**
Active	248	98.0%	297	99.7%		72	69.9%	126	98.4%	
Died	5	2.0%	1	0.3%		31	30.1%	2	1.6%	
**Programme outcome**					0.697					**0.002**
Active	248	92.9%	297	93.7%		72	60.5%	126	77.3%	
Died or lost to follow-up	19	7.1%	20	6.3%		47	39.5%	37	22.7%	
**Suppression at last viral load test**					**< 0.001**					**< 0.001**
Suppressed	134	46.5%	251	72.3%		46	63.0%	107	85.6%	
Not suppressed	154	53.5%	96	27.7%		27	37.0%	18	14.4%	

ART, antiretroviral therapy; CPT, cotrimoxazole preventive therapy; IPT, isoniazid preventive therapy; WHO, World Health Organization.

Data are *n* (%) unless otherwise indicated. Total value differs between variables because of missing data.

Statistically significant differences are shown in bold.

**FIGURE 4:**
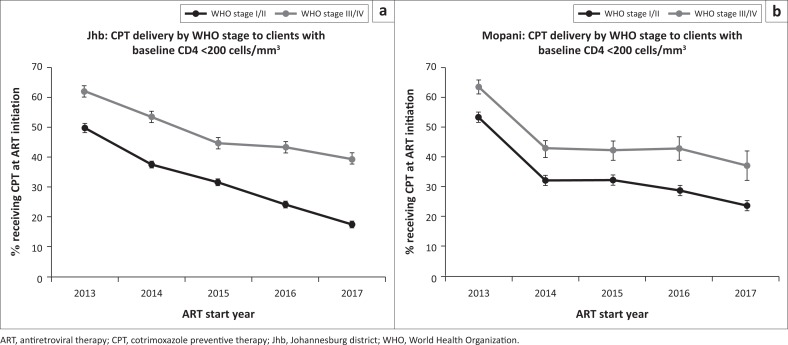
Delivery of cotrimoxazole preventive therapy over time to clients with baseline CD4 counts < 200 cells/mm^3^ by clinical stage in (a) Johannesburg district and (b) Mopani district.

Delivery of CPT at treatment initiation in 2017 was poor – in Johannesburg and Mopani, only 23% and 26% of clients with baseline CD4 counts < 200 cells/mm^3^ are documented as having received CPT, respectively (Table 2). Nevertheless, as expected, significantly more clients with low CD4 received CPT compared to clients with CD4 counts > 200 in both districts (*p* < 0.001) in line with national guidelines.^9^ In the last 5 years (2013–2017), delivery of CPT to clients initiating ART with baseline CD4 counts < 200 cells/mm^3^ has declined in both Johannesburg and Mopani districts (Figure 4). Furthermore, a consistently higher proportion of clients presenting with advanced disease (WHO stage III or IV) received CPT compared to those presenting with WHO stage I or II disease across all 5 years.

Among clients initiating ART at primary care clinics in 2017, mortality was significantly higher and viral suppression was significantly lower among clients with baseline CD4 counts < 200 cells/mm^3^ compared to those with higher baseline counts (*p* < 0.001), with similar findings evident in hospitals (Table 2). The high mortality in Mopani hospitals was largely from a single facility with 16 deaths, although the increased mortality in clients with low CD4 counts in Mopani hospitals remained significant even when this hospital was removed from the analysis (*p* < 0.001).

## Discussion

This study of routine TIER.Net data demonstrates an increase in mean and median baseline CD4 counts over time, in line with South African guidelines that have repeatedly raised the CD4 cut-off for ART initiation^5,8,9,10^ and in agreement with previous findings in South Africa and other countries.^2,16,24,25,26^ However, the improvement in these population measures is masking a high-risk group of clients with low CD4 counts who only initiate treatment once they have advanced HIV disease. Although the proportion of clients initiating ART with CD4 counts < 100 and < 200 cell/mm^3^ declined in the initial years of the ART programme, as has been previously demonstrated from 2001 to 2012,^12^ there has been little change in recent years, with the proportion of clients with low baseline CD4 counts remaining concerningly high. This is unexpected in the context of UTT, but it confirms findings from a recent South African study of laboratory data which also noted little change in the proportion of adult clients initiating ART with CD4 counts < 200 cells/mm^3^ between 2012 and 2016, ranging from 32.9% to 34.8% nationally.^27^ This is also a challenge in other low- and middle-income countries, with over 30% of clients initiating treatment with CD4 counts < 200 cells/mm^3^ despite generally widespread availability of ART.^24,25^ These clients, who may either be ART-naïve or may have previously defaulted from the ART programme, represent a considerable burden of increased medical costs, risk of transmission and HIV morbidity.^27^ Interventions to address HIV incidence as well as barriers to entering care are essential.

Mortality was found to be significantly higher in clients with baseline CD4 counts < 200 cells/mm^3^ compared to those with higher CD4 counts, as has been well established in South Africa and other settings.^3,16,17,28^ Interestingly, it has been shown that standardised mortality rates which take background mortality into account are similar for all CD4 strata by 4 years after ART initiation, suggesting that baseline immunological status impacts mortality primarily at shorter durations of ART.^2^ It is specifically at these early time periods after ART initiation that there is a notably high mortality rate, as has been demonstrated in this study, specifically among clients with baseline CD4 counts <200 cells/mm^3^. Other studies in South Africa and sub-Saharan Africa have similarly demonstrated high mortality rates in the first year after starting ART, with up to 26% of patients dying in the first year of treatment.^3,29^ There is a strong association between early mortality and the degree of immunodeficiency at ART start; therefore, strategies to reduce mortality must address ‘up-stream’ entry points into the treatment cascade, including earlier diagnosis of HIV and improved longitudinal care pre-ART.^29^

Although clients with low baseline CD4 counts are at increased risk of death, mortality rates have decreased in recent years of the ART programme in this and other studies.^2,16,17,26^ Overall mortality in South Africa has declined in recent years^30^ and the gains in life expectancy have been largely attributed to a decline in HIV-related mortality rates.^31^ This decline can be ascribed to improved accessibility to HIV services with the implementation of nurse-managed care,^11^ as well as improved quality of care because of enhanced drug regimens. This study demonstrated significantly lower mortality among clients with CD4 counts < 200 cells/mm^3^ who initiated ART in more recent years when an FDC was in use compared to those initiating treatment in previous years, with the highest mortality in years when guidelines recommended the use of stavudine which has a relatively unfavourable toxicity profile.^32^ Antiretroviral therapy using an FDC has been shown in a European study to improve both adherence and quality of life of HIV-infected patients, while maintaining virological and immunologic efficacy.^33^ In general, the simplification of ART with an FDC is associated with improved adherence because of decreased pill burden, reduced risk of resistance owing to the inability of patients to take partial regimens, reduced risk of treatment or prescription errors and lower risk of hospitalisation,^34,35^ supporting the assertion that FDCs may contribute to reduced mortality rates. However, in the context of overall ART programme performance, it is important to note that LTFU appears to be increasing in more recent years, possibly because of the expansion of treatment programmes in the absence of sufficient resources.^26,36^ It is important that overall programme performance is monitored in order to minimise LTFU and the knock-on effect that this could have on mortality.

To improve outcomes of the HIV programme, interventions should focus on clients most likely to present late for HIV care, including, as expected, clients with advanced clinical stage of HIV disease and those in hospitals. Interventions such as community-based strategies or home-based HIV testing should also target men,^37,38^ in agreement with previous studies in South Africa and other low- and middle-income countries which demonstrate late presentation of men for both HIV diagnosis and treatment.^39,40,41,42,43^ This delayed health seeking may contribute to the growing gap between male and female life expectancy, which has doubled from 2003 to 2011.^44^ Reasons for the disparity are likely to include reluctance of men to seek HIV care because of attitudinal barriers, HIV stigma, perceptions of masculinity and cultural practices,^45,46^ as well as the scale-up of prevention of mother-to-child transmission (PMTCT) services,^47^ which have increasingly engaged women in care. This study found that a higher proportion of pregnant women aged 15–50 years had baseline CD4 counts ≥ 200 cells/mm^3^ compared to women who were not pregnant during ART initiation, suggesting that PMTCT services play a role in linking women to timely HIV treatment. This is supported by findings of an association between PMTCT services and higher cohort median CD4 count, as well as reduced risk of ART initiation with advanced disease.^41,42,48^ In addition, late presentation for ART initiation was found to be linked with older age in this study. Mixed findings regarding the association between age and late presentation for care have been reported in the literature, with one study finding no significant association,^39^ others finding a decreased risk of late presentation for HIV care in older clients^41,42^ and yet others demonstrating an increased risk of initiating ART with low CD4 in older clients.^25,40^ The finding of this study that older age is linked to late presentation for care is supported by a recent study of South Africans aged over 50 years who reported seeking HIV testing only once they were symptomatic and referred by a provider or if a partner was diagnosed as infected.^49^ Furthermore, older men in sub-Saharan Africa have been found to be less aware of and knowledgeable about HIV prevention compared to younger men, with increased risk behaviours in some settings.^50^ Educational initiatives and HIV testing strategies that focus on men and the older population are thus required.

An additional intervention to improve outcomes of the HIV programme is delivery of CPT to high-risk clients presenting for HIV care with low baseline CD4 counts, as recommended by the WHO.^51^ It has been well established that CPT significantly reduces mortality in clients with low baseline CD4 counts,^52,53^ and it is therefore concerning that delivery of this intervention appears to be declining over time, possibly reflecting competing demands on healthcare providers as the ART programme expands. Interestingly, a consistently higher proportion of clients with WHO stage III or IV disease received CPT compared to clients presenting with WHO stage I or II disease, indicating that healthcare providers are more likely to give CPT to clients who appear ill. This is concerning, as clients with immunosuppression who require CPT may not present with advanced disease, as seen in this and other studies,^54^ and delivery of CPT may therefore be missed in these clients. This is likely exacerbated in the era of UTT, as baseline CD4 counts which would alert healthcare providers to the need for CPT are not required at the time of ART initiation; delivery of CPT therefore relies on the healthcare provider later checking the CD4 results and subsequently initiating CPT if required, increasing the chance that CPT may be overlooked. Interventions to increase awareness of the importance of baseline CD4 testing and delivery of CPT to clients with low baseline CD4 counts and/or advanced clinical disease are warranted.

The urban and rural districts analysed in this study had comparable findings in terms of overall trends in population CD4 counts, the proportion of clients initiating ART with CD4 < 200 cells/mm^3^ in 2017 and characteristics of clients with low baseline CD4 counts. However, mortality was markedly higher in rural Mopani than in Johannesburg. This highlights the inferior quality of care in the rural district, likely because of poor access to doctors as has been previously described in rural settings, as well as the deficit in specialist medical care in rural provinces of South Africa.^55,56,57^ In addition, characteristics of rural settings, such as lower levels of formal education, are also known to be associated with higher mortality.^58^ While urban settings need to be aware of the rate of programme expansion owing to the increased risk of attrition associated with rapid expansion of ART services,^36^ efforts in rural settings need to focus on quality of care, perhaps by employing mobile units that are staffed by doctors or by providing training for primary healthcare staff.

This study demonstrated the rich source of data available in TIER.Net. The analysis was performed using data spanning over a decade during which there were substantial guideline changes in the ART programme, producing a robust and realistic analysis. On the other hand, this study has a number of limitations. Firstly, TIER.Net was introduced in South Africa in 2010, necessitating the back-capture of data from the ART programme prior to this time. There may have been challenges with the quality of the back-captured data, which may have led to an underestimation of the absolute numbers of clients on ART, including those who died, although we believe that the proportions are representative. Secondly, not all clients in TIER.Net could be included in the analysis because of missing baseline CD4 counts. Thirdly, the study was not designed to follow up clients who were LTFU from the ART programme and it is therefore unclear how many of these clients died. The Kaplan–Meier analyses may therefore underestimate mortality. Finally, although these data are representative of urban and rural areas in South Africa, they should be generalised to other settings with caution, particularly because only two districts were included in the analysis.

## Conclusion

In conclusion, this study of routine programme data demonstrates rapid expansion of South Africa’s adult ART programme since 2004. Although median CD4 count has increased over time, this population measure is masking a high-risk group of clients who present late for HIV care with low baseline CD4 counts. Despite improvements in mortality over time, these clients are still at risk of increased mortality, particularly in rural settings. Therefore, targeted interventions are urgently warranted to improve early engagement in care and clinical management, with a specific focus on men, older clients and those presenting at hospitals. Ongoing monitoring of the routine programme coupled with targeted interventions will ensure continued improvements in the outcomes of these high-risk individuals.
